# An IBD-associated pathobiont synergises with NSAID to promote colitis which is blocked by NLRP3 inflammasome and Caspase-8 inhibitors

**DOI:** 10.1080/19490976.2022.2163838

**Published:** 2023-01-19

**Authors:** Raminder Singh, Valerio Rossini, Stephen R. Stockdale, Gonzalo Saiz-Gonzalo, Naomi Hanrahan, Tanya D’ Souza, Adam Clooney, Lorraine A. Draper, Colin Hill, Ken Nally, Fergus Shanahan, Stefan Andersson-Engels, Silvia Melgar

**Affiliations:** a APC Microbiome Ireland, University College Cork, Cork, Ireland; b Department of Medicine, School of Medicine, University College Cork, Cork, Ireland; c School of Biochemistry and Cell Biology, University College Cork, Cork, Ireland; d School of Microbiology, University College Cork, Cork, Ireland; e Irish Photonics Integration Centre, Tyndall National Institute, Cork, Ireland; f Department of Physics, University College Cork, Cork, Ireland

**Keywords:** AIEC, piroxicam, inflammatory bowel disease, cell death, IL10^−/−^ mice, inflammasome

## Abstract

Conflicting evidence exists on the association between consumption of non-steroidal anti-inflammatory drugs (NSAIDs) and symptomatic worsening of inflammatory bowel disease (IBD). We hypothesized that the heterogeneous prevalence of pathobionts [e.g., adherent-invasive *Escherichia coli* (AIEC)], might explain this inconsistent NSAIDs/IBD correlation. Using *IL10^−/−^* mice, we found that NSAID aggravated colitis in AIEC-colonized animals. This was accompanied by activation of the NLRP3 inflammasome, Caspase-8, apoptosis, and pyroptosis, features not seen in mice exposed to AIEC or NSAID alone, revealing an AIEC/NSAID synergistic effect. Inhibition of NLRP3 or Caspase-8 activity ameliorated colitis, with reduction in NLRP3 inflammasome activation, cell death markers, activated T-cells and macrophages, improved histology, and increased abundance of *Clostridium* cluster XIVa species. Our findings provide new insights into how NSAIDs and an opportunistic gut-pathobiont can synergize to worsen IBD symptoms. Targeting the NLRP3 inflammasome or Caspase-8 could be a potential therapeutic strategy in IBD patients with gut inflammation, which is worsened by NSAIDs.

## Introduction

Inflammatory bowel disease (IBD) is a chronic inflammatory state of the gastrointestinal tract, including Crohn’s disease (CD) and Ulcerative colitis (UC). The etiology of IBD is unknown, but the collective evidence indicates that a dysregulated immune response to commensal enteric microorganisms (one of the hallmarks of IBD) leads to uncontrolled chronic inflammation in the genetically susceptible host.^1,2^

Non-steroidal anti-inflammatory drugs (NSAIDs) are commonly used for various inflammatory conditions, including IBD-associated extraintestinal manifestations. However, mucosal injury and ulcers are common adverse effects of NSAID use and are therefore believed to exacerbate inflammation in patients with IBD.^3^ However, the current literature on the association between NSAIDs and IBD is inconclusive.^4,5^ Evidence to date suggests that the gut microbiota might play a critical role in NSAID-promoted inflammation.^6^ For example, germ-free rats are resistant to NSAID-induced intestinal damage^7^ and an increase in the relative-abundance of potentially harming bacteria such as *Rikenellaceae, Pseudomonadaceae, Propionibacteriaceae*, and *Puniceicoccaceae* was observed in individuals taking ibuprofen.^8^ Furthermore, an increased expansion of Gram negative taxa such as *Escherichia/Shigella, Bilophila, and Bacteroides* was reported in the ileum of rats with indomethacin-induced enteropathy.^9^ In addition, consumption of a *Bifidobacterium* strain reduced NSAID-induced ulcerations in healthy volunteers.^10^ No reports on how NSAIDs might affect the microbiota of patients with IBD or how the presence of certain IBD-associated bacteria may affect the response of the patients when treated with NSAIDs have been found in the literature.

Genetic background and environmental factors can contribute to disease triggered by pathogenic commensals, so-called pathobionts.^11^ Adherent-invasive *Escherichia coli* (AIEC) is a gut pathobiont widely prevalent in IBD patients in Western countries, up to 10% and 62% in patients with UC and CD, respectively.^12,13^ AIEC has been reported to trigger inflammation in genetically predisposed mice and even promote fibrosis in chemically induced colitis.^14^ A recent study reported that AIEC might be associated with the early phase of recurrence in patients with CD,^15^ further indicating their potential contribution to CD pathogenesis.

NOD-like receptors (NLRs) are an important family of pathogen recognition receptors (PRRs) that sense pathogen and commensal associated molecular patterns (PAMPs), or danger associated molecular patterns (DAMPs), leading to co-ordinated innate and adaptive immune responses. One of the most intensely studied members of this NLR family is NLRP3, whose activation leads to the formation of the NLRP3 multiprotein inflammasome complex. Formation of this complex activates caspase-1 and subsequent cleavage of downstream substrates such as the effector inflammatory cytokines, pro-IL1β and pro-IL18.^16^ Activation of NLRP3 has been reported in pre-clinical models of intestinal infection and colitis, in human IBD tissue and by AIEC in macrophages.^17,18^ Genome-wide association studies have identified an SNP in the region of the NLRP3 gene possibly contributing to disease susceptibility in CD, and a recent study reported a loss-of-function mutation in the CARD8 domain, causing NLRP3 activation and CD.^19^ NLRP3 has also been shown to play a crucial role in NSAID-induced enteropathy.^20^

One of the hallmarks of chronic inflammation is the increased death (apoptosis) of intestinal epithelial cells in patients with IBD.^21^ One initiator caspase, which acts as a central regulator of the crosstalk and plasticity between multiple cell death pathways (apoptosis, necroptosis, and pyroptosis) and inflammation, is Caspase-8.^22^ Various studies have shown the role of Caspase-8 in intestinal inflammation.^23,24^ Activation of death receptors (e.g., TNFR), Toll-like receptors (TLR3/-4), the z-form nucleic acid sensor (ZBP1), or the intracellular RNA sensor RIG-I leads to the recruitment of Caspase-8 to the intracellular signaling complex, followed by its autoproteolytic cleavage and activation. Active caspase-8 cleaves its substrate caspase-3/7, initiating apoptosis. In addition, active caspase-3/7 can also cleave poly(ADP-ribose) polymerase (PARP), resulting in aberrant DNA repair and apoptosis.^22,25^ Active Caspase-8 inhibits necroptosis by suppressing the function of receptor-interacting protein kinase 1 (RIPK1) and RIPK3, to inhibit the hosphorylation of the pore-forming mixed lineage kinase domain-like (MLKL) protein.^2^

Another cell death mechanism provoked by inflammation and microbial infections, e.g., *Salmonella*, is pyroptosis. This cell death can be induced by the canonical activation of Caspase-1 or via the non-canonical caspases, Caspase-11 (mice) and Caspase-4/5 (humans),^2^ leading to the cleavage of its main executor gasdermin D (GSDMD). Interestingly, Caspase-8 has been shown to cleave GSDMD leading to pyroptosis.^26^ Caspase-8 can act both upstream and downstream of NLRP3 inflammasome activation. On the one hand, it triggers NLRP3 activation via both its enzymatic activity and scaffolding function.^27–30^ On the other hand, NLRP3 can regulate Caspase-8 activation in epithelial cells independent of Caspase-1 activation or cytokine production.^31^

Based on the collected evidence to date, we hypothesized that the apparent inconsistency in reports regarding the impact of NSAIDs on IBD disease activity could be explained by a dysregulated microbiota composition and the presence of pathobionts such as AIEC in these patient cohorts. In this study, we used interleukin-10 deficient (*IL10^−/−^*) mice, a pre-clinical IBD model used to mimic the genetic predisposition in IBD, to test our hypothesis. We show that AIEC and NSAIDs combine to induce/exacerbate colitis and cell death via the NLRP3 inflammasome and Caspase-8 with evidence of NLRP3-Caspase-8 cross-talk contributing to this overall phenotype. This provides new insights into how NSAIDs in the presence of an opportunistic gut pathobiont could worsen IBD symptoms.

## Results

### AIEC-mediated sensitization of IL10^−^^/-^ mice to piroxicam induced-inflammation is associated with activation of the inflammasome, apoptosis, and pyroptosis

**Figure 1.**
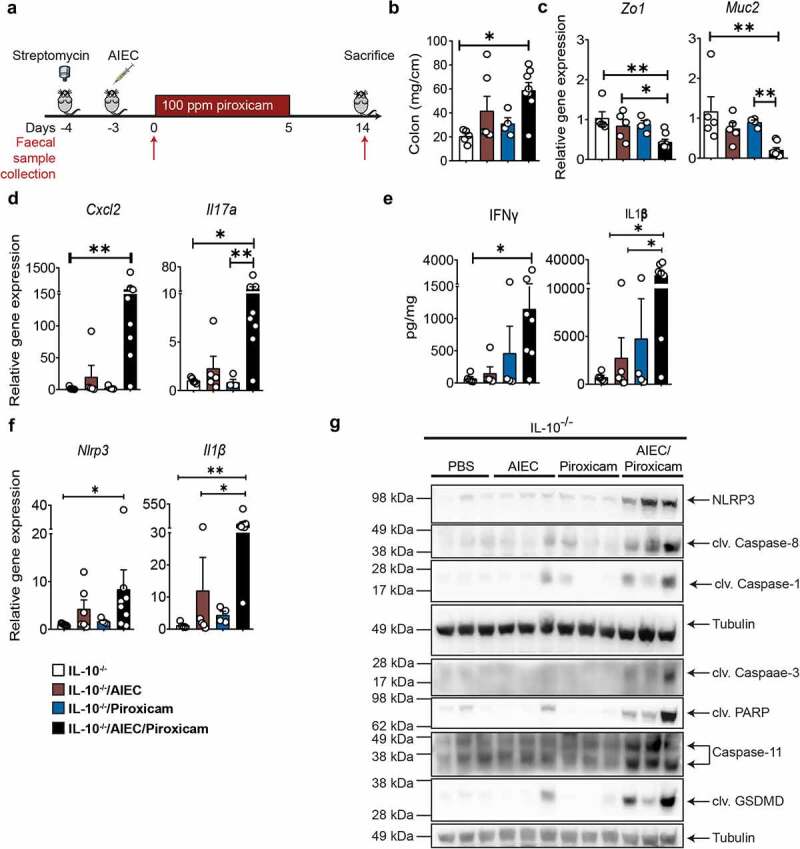
*IL10* *^−/−^* mice colonized with AIEC and fed piroxicam develop colitis associated with activation of N LRP 3 inflammasome and Caspase-8. (a) Study design. *IL10* ^*−/−*^ mice were given streptomycin (5 g/L) in drinking water *ad libitum* for 24 hrs, followed by oral gavage with approx. 10 ^9^ AIEC colony forming units (CFU), 100 ppm of piroxicam homogenized in normal chow for days followed by normal chow until day 14. Red arrows indicate timepoints for fecal collection. (b) Distal colon weight. (c) Real- time (RT)- qPCR for colonic epithelial and (d) inflammatory markers. (e) Protein levels of colonic inflammatory cytokines and chemokines. (f) RT- qPCR for colonic *Nlrp3* and *Il1b*. (g) Western blot of markers of NLRP3 inflammasome, Caspase-8, apoptosis and pyroptosis markers. n = 3–8/group for RT- qPCR, protein, western blot, and necroscopy. Data are presented as mean ± SEM. Significance was determined using one- way ANOVA with Bonferroni or Kruskal–Wallis test with Dunn’s multiple comparison test, respectively. * p < .05; ** p < .01; *** p < .001.

To test our hypothesis, we examined the ability of AIEC-HM605, a strain of AIEC isolated from colonic biopsies of a patient with CD,^32^ to sensitize *IL10^−/−^* mice to NSAID (piroxicam) induced inflammation. We developed a model (Figure 1a) whereby initial pre-treatment of *IL10^−/−^* mice with streptomycin facilitated colonization with AIEC^33^ (Figure S1C). Subsequent feeding of these mice with 100 ppm of piroxicam in regular chow for 5 d followed by 9 d of normal chow resulted in colitis (Figure 1 and Figure S1). The initial colonization of AIEC dropped from 10^9^ to 10^4^ cfu/g at day 7, followed by a stable AIEC colonization throughout the study (Figure S1C). A similar degree of AIEC colonization (10^4^ cfu/g) was seen in the colon tissue of animals in the AIEC and AIEC/piroxicam groups at day 14 (data not show). Similar changes in disease activity index and body weight loss were observed in the AIEC/piroxicam group, with a higher non-significant body weight loss at day 14 when compared to AIEC or piroxicam only groups (Figure S1A-B). In line with other models of colitis,^34^ only the mice colonized with AIEC and subsequently treated with NSAID had shorter and heavier colons compared to animals exposed to piroxicam or AIEC alone (Figure 1b). This was accompanied by a loss of colonic epithelial integrity indicated by a significant reduction in expression of colonic *Zo1* and *Muc2* genes (Figure 1c) and a significant induction in the expression of inflammatory genes and proteins (*Cxcl2, Il17a, Ifng* and IFNγ, IL1β, and mKC, Figure 1d-e, S1D-E).

Because the NLRP3 inflammasome has previously been shown to be activated by AIEC strains *in vitro* and in NSAID enteropathy,^18,20^ we analyzed its activation by AIEC/NSAID in our model. Indeed, a significant upregulation of colonic NLRP3, caspase-1, and its downstream target IL-1β was revealed at the gene expression and protein level in the AIEC/piroxicam combination group but not in mice colonized only with AIEC or administered piroxicam alone (Figure 1e-g). We also observed Caspase-8 cleavage, which is indicative of Caspase-8 activation in the AIEC/piroxicam combination group (Figure 1g). To confirm Caspase-8 activation, we analyzed cleavage of its downstream target NEDD4-binding protein 1 (N4BP1).^35^ Indeed, cleaved N4BP1 was only detected in AIEC/piroxicam combination group (Figure S1F). Since Caspase-8 can induce apoptosis in part by cleaving and activating Caspase-3,^36^ we analyzed and detected the cleaved form of Caspase-3 in the AIEC/piroxicam group when compared to AIEC or piroxicam only groups (Figure 1g). Consistent with this, cleaved PARP was also detected in the AIEC/piroxicam group (Figure 1g). No activation of necroptosis, which was analyzed by western blotting for phospho-MLKL or RIPK1, was seen in any of the groups (data not shown). When we examined the expression of the main executor and biochemical marker of pyroptosis – cleaved GSDMD – we found it was only expressed in the AIEC/piroxicam group (Figure 1g), indicating a synergistic activation of pyroptosis by AIEC/piroxicam.

Collectively, these observations validate our initial hypothesis indicating that AIEC colonization in a genetically predisposed host renders them susceptible to NSAID-induced inflammation. This inflammation is accompanied by activation of the inflammasome, apoptosis, and pyroptosis.

### Colonization with AIEC and piroxicam treatment provoked modest alterations of the gut microbiome in IL10^−^^/-^ mice

To determine the impact of piroxicam and/or AIEC exposure on the gut microbiome, we conducted 16S rRNA gene analysis on fecal samples collected at day 0 and 14 (Figure 1a). Across treatment groups, an increase in intra-sample alpha-diversity was observed over time (Figure S2A), suggesting a gradual recovery of mice microbiomes following Streptomycin treatment. While there is a significant (p < .05) increase in alpha-diversity observed in AIEC, and AIEC/piroxicam treated mice between day 0 and day 14 (Figure S2A), this trend was non-significant after false-discovery rate (fdr) correction.

Visually, the ordination of mice 16S rRNA composition shows that beta-diversity differences are greater in mice colonized only with AIEC and/or treated with piroxicam compared to PBS-treated controls (Figure S2B). Permutational multivariate analysis of variance (PERMANOVA) tests were performed on inter-sample beta-diversity distances to discern the variables significantly contributing to data dispersion (i.e., variance). This analysis revealed that the days post-piroxicam variable accounted for 14.9% of the 16S rRNA data variance (Figure S2B).

The compositional bar plots on 16S rRNA data show only taxa with a total aggregated relative abundance across all mice greater than 0.1% (Figure S2C). As expected, *Escherichia/Shigella* are detected at day 0 (i.e., 4 d post-AIEC colonization) of the relevant treated animals. However, significant differences in other individual 16S microbial taxa were not observed between mice over time after fdr correction, although some alterations were observed, e.g., increased abundance in *Alistipes* and *Bacteroides* in AIEC/piroxicam group, changes often associated with murine colitis.^37^ When individual cecal short chain fatty acids (SCFAs) levels were examined, a general but non-significant increase in propionate and reduction in acetate was observed in the AIEC/piroxicam group (Figure S1G).

Collectively, the data showed that AIEC and/or piroxicam treatment only had modest effects on gut microbiome composition and SCFAs in contrast to other colitis stimuli.^37^

### Caspase 8 and NLRP3 inhibitors ameliorate NSAID induced epithelial alterations and inflammation in AIEC colonized mice

**Figure 2.**
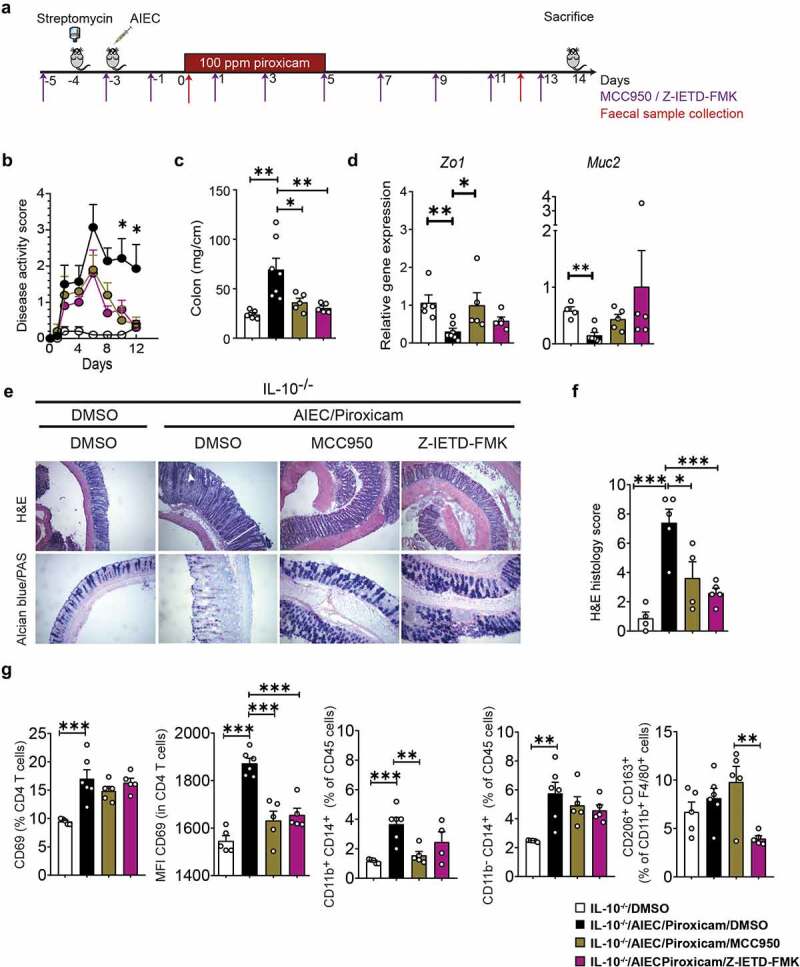
Caspase-8 and NLRP3 inhibitors improve AIEC induced epithelial and immune alterations in *IL10^−/−^* mice fed with piroxicam. (a) Study design. Mice were injected intra-peritoneally with NLRP3 inhibitor (MCC950, 20 mg/kg) or Caspase-8 inhibitor (Z-IETD-FMK, 10 mg/kg) starting at day −5 and every second day as indicated with purple arrows, followed by euthanasia at day 14. Red arrows indicate timepoints for fecal collection. (b) Disease activity index. (c) Colon weight. (d) RT-qPCR of colonic epithelial genes. (e) Representative Hematoxylin and Eosin (H&E) and alcian blue (AB)/PAS staining of distal colon sections. In AB/PAS staining, Goblet cells are stained in dark purple color. White arrow indicates crypt abscess and white line indicate crypt hyperplasia in H&E stained sections. (f) Histology score. n = 4–7/group. (g) Isolated spleen T-cells (CD4 and CD69) and macrophages (CD11b, CD14, CD163, CD206) were immunophenotyped by Fluorescence-activated cell sorting (FACS) after gating on CD45 (Figure S2A-C) and expressed as MFI or percent of specific cell populations. n = 5–6/group. Significance was determined using one-way ANOVA with Bonferroni or Kruskal–Wallis test with Dunn’s multiple comparison test, respectively. * p < .05; ** p < .01; ***p < .001.

**Figure 3.**
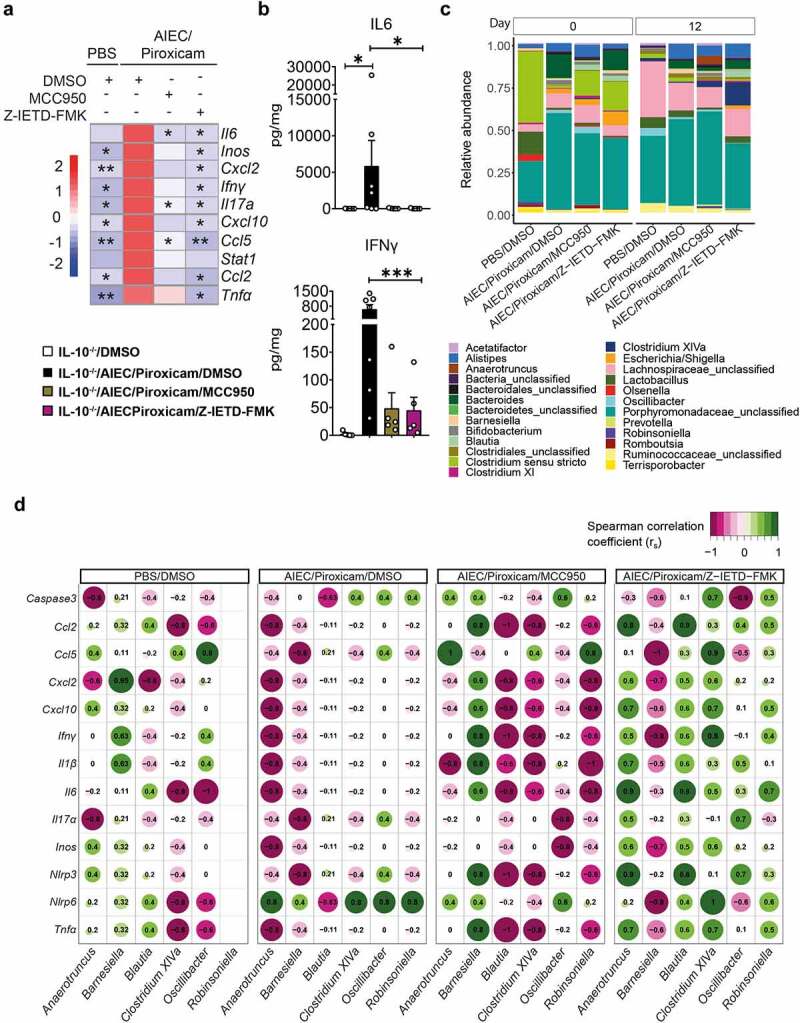
Caspase-8 and NLRP3 inhibitors have a differential effect on host–microbe interactions in AIEC-colonized *IL10^−/−^* mice treated with piroxicam. (a) Heat map of colonic gene inflammatory expression. AIEC-piroxicam/DMSO treated group was used as control for statistical analysis. (b) Protein level of colonic cytokines and chemokines n = 5–7/group. Data are presented as mean ± SEM. (c) Fecal samples were collected for 16S rRNA analysis at days 0 (upon piroxicam feeding) and 14 (end of trial). Data is presented as fecal bacteria relative-abundance at genus-level. (d) Spearman correlation between host inflammatory gene expression and specific microbial genera at day 14. n = 4–9/group. For (a) and (b), significance was determined using one-way ANOVA with Bonferroni or Kruskal–Wallis test with Dunn’s multiple comparison test, respectively. * p < .05; ** p < .01; ***p < .001.

To further investigate the role of NLRP3 and Caspase-8 in our model, *IL10^−/^*^−^ mice were administered the selective NLRP3 inhibitor, MCC950,^38^ and the Caspase-8 inhibitor Z-IETD-Fmk,^39^ with the treatment of mice starting before AIEC and piroxicam exposure (Figure 2a). Both inhibitors significantly reduced the disease activity index and colon weight of animals administered AIEC and piroxicam (Figure 2b-c). These inhibitors improved the loss of epithelial integrity, as shown by the recovery of colonic *Zo1* and *Muc2* gene expression (Figure 2d). Similarly, histology analysis revealed a significant recovery of epithelial structure represented by less elongated crypts and reduced presence of crypt abscesses (H&E staining) and an increase in mucus-secreting goblet cells (Alcian blue/PAS staining) (Figure 2e-f). Furthermore, inhibition of NLPR3 or Caspase-8 reduced the infiltration of inflammatory cells into the lamina propria (Figure 2f). By flow cytometry, we also observed that the Caspase-8 inhibitor significantly reduced activated T-cells (CD4^+^ CD69^+^) in the spleen, while NLRP3 inhibition reduced both activated T-cells and monocytes/macrophages (CD11b^+^ CD14^+^ and CD11b^−^ CD14^+^), while M2 macrophages (CD206^+^CD163^+^) were increased (Figure 2g and Figure S3A-C). A similar trend in activated T-cells was seen in the mesenteric lymph nodes treated with either of the inhibitors (data not shown). These changes in immune cell populations were accompanied by a reduction in colonic gene expression of acute inflammatory cytokines (*Il6, Cxcl2, and Inos)*; macrophage activation markers (*Tnfa, Ccl2*) and T-cell markers (*Ccl5, Cxcl10, Il17a,* and *Ifng*) (Figure 3a); all of which were initially altered in the AIEC/piroxicam group (Figure 3a, 1d-e, and S1D-E). Similarly, a reduction in protein levels of IL-6 and IFN-γ was also observed in mice treated with either inhibitor compared to AIEC/piroxicam mice (Figure 3b). Together, these findings suggest that activation of the NLRP3 inflammasome and Caspase-8 may alter epithelial cell biology and barrier integrity through increased inflammatory cell death exacerbating inflammation which is characterized by the presence of activated T-cells and macrophages in NSAID-exposed *IL10^−/−^* mice pre-colonized with AIEC.

### Differential microbiome–host interactions are associated with effects of NLRP3 and Caspase-8 inhibition

Next, we investigated the effects of the small-molecule inhibitors targeting NLRP3 (MCC950) and Caspase-8 (Z-IETD-FMK) on bacterial composition in fecal samples collected on day 0 and 12 from *IL10^−/−^* mice with colitis induced by AIEC/piroxicam (Figure 2a). No significant changes were detected in 16S rRNA alpha-diversity between the different groups or over time (Figure S3D). When we investigated the variance associated with sample beta-diversity differences (Figure S3E), the variance attributable to days post piroxicam was 12.3%, while the treatment grouping variable was associated with an 11.4% variance. The interaction of these two variables was 8.8% (p-value = 0.011).

To avoid over-cluttering of the compositional bar plot of AIEC/piroxicam inflamed mice versus Caspase-8/NLRP3 inhibitor groups, only taxa with a total aggregated relative abundance of 0.2% are shown (Figure 3c). Visually, the 16S compositions at day 0 showed a higher abundance of *Clostridium sensu stricto* in the inhibitor treated groups, while at day 12, *Clostridium* cluster XIVa abundance was seen in mice treated with both inhibitors. However, these did not reach statistically significant after fdr correction, potentially due to the small group size comparisons (Figure 3c).

Interestingly, the rank order Spearman correlations of microbial taxa versus inflammatory markers showed contrasting results with regard to the inhibitor treatment (Figure 3d). For putative *Anaerotruncus, Barnesiella, Blautia, Clostridium XIVa, Oscillibacter*, and *Robinsoniella*, the correlation coefficients were almost exactly opposite in the NLRP3 (MCC950) and Caspase-8 (Z-IETD-FMK) inhibitor-treated groups. These microbial correlation differences occur despite both inhibitors similarly dampening the colonic epithelial and inflammatory gene expression profile (Figure 3a).

When the individual cecal SCFA levels were examined, a non-significant increase in propionate was observed in the Caspase-8 and NLRP3 inhibitor-treated groups compared to the AIEC/piroxicam group (Figure S3F).

Overall, these data indicate that NLRP3 and Caspase-8 inhibitors target-specific microbiome–host interactions without provoking dramatic alterations on microbial taxa.

### Caspase-8 and NLRP3 inhibitors block inflammasome activation, apoptosis, and pyroptosis in AIEC colonized and NSAID exposed mice

**Figure 4.**
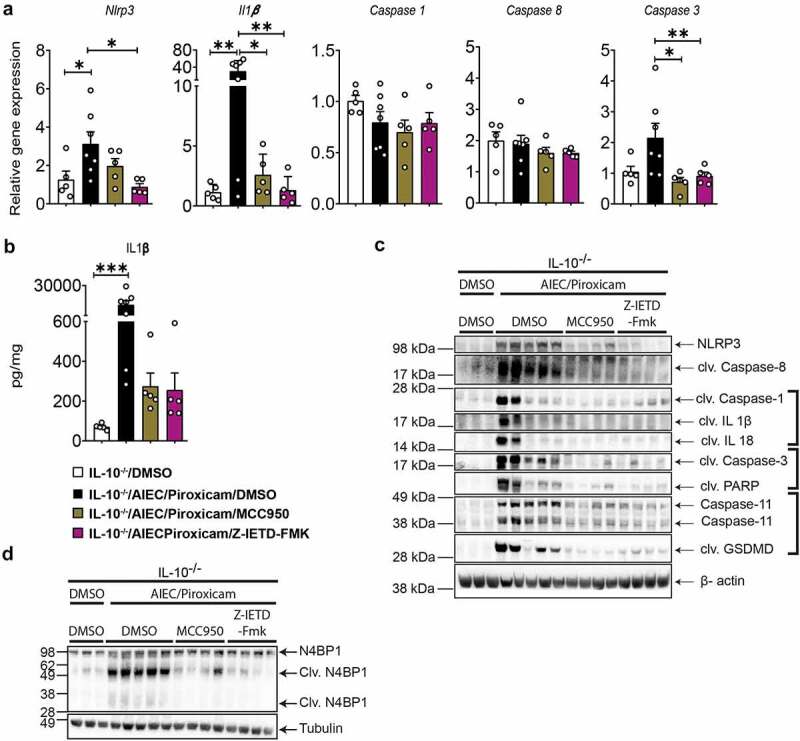
Caspase-8 and NLRP3 inhibitors reduced NLRP3 inflammasome, apoptosis and pyroptosis in AIEC colonized *IL10^−/−^* mice after piroxicam feeding. (a) RT-qPCR of colonic NLRP3 inflammasome, and caspases. (b) IL-1β protein expression. (c) Western blot of Caspase-8, inflammasome and cell death markers. (d) Western blot of N4BP1. n = 3–7/group. Data are presented as mean ± SEM. Significance was determined using one-way ANOVA with Bonferroni or Kruskal–Wallis test with Dunn’s multiple comparison test, respectively. * p < .05; ** p < .01; *** p < .001.

AIEC/piroxicam combination in *IL10^−/−^* mice resulted in activation of the inflammasome, apoptosis, and pyroptosis (Figure 1g). When assaying the expression of genes associated with inflammasome activation and apoptosis in mice administered either the NLRP3 or Caspase-8 inhibitors, no changes were observed at the gene expression levels of *Caspase 1* and *Caspase 8*, while *Il1b, Nlpr3*, and *Caspase 3* were significantly reduced when compared to AIEC/piroxicam group (Figure 4a-b). In support of these findings, western blot analysis showed decreased expression of the inflammatory active cleaved Caspase-1, IL-1β, and IL-18, the pro-apoptotic markers – cleaved Caspase-3 and PARP, and the pyroptotic markers – cleaved Caspase-1, Caspase-11, and GSDMD, in the inhibitor-treated groups compared to AIEC/piroxicam combination group (Figure 4c). As expected, both the NLRP3 inhibitor (MCC950) and the Caspase-8 inhibitor (Z-IETD-FMK) reduced the expression of their own target proteins, and both inhibitors decreased the expression of the same biochemical markers of inflammasome activation, apoptosis, and pyroptosis (Figure 4c). Interestingly, western blot analysis indicated that both Caspase-8 and NLRP3 can potentially cross-regulate each other. Caspase-8 inhibition reduced NLRP3 both at the gene and protein levels, while NLRP3 inhibition reduced the expression of active cleaved Caspase-8. Several studies have reported that Caspase-8 may act upstream of NLRP3, by inducing NLRP3 activation via GSDMD-mediated K^+^-efflux.^29^ To confirm the involvement of Caspase-8 and its interaction with NLRP3, we assessed the expression of N4BP1, a downstream substrate of Caspase-8.^35^ As previously shown in Figure S1F, N4BP1 was cleaved in AIEC/piroxicam combination group, but not in the NLRP3 inhibitor group (Figure 4d), suggesting a role for NLRP3 in Caspase-8 regulation in our model. In line with our expectations, no N4BP1 cleavage was detected in the Caspase-8 inhibitor group (Figure 4d), confirming Caspase-8 inhibition. The downregulation in *Nlrp3* gene expression observed in Caspase-8 inhibitor group could be explained by the regulation of NF-κB by Caspase-8.

## Discussion

In this study, we investigated if the presence of an IBD-associated pathobiont (AIEC)^13,40^ would exacerbate intestinal inflammation and worsen symptoms triggered by subsequent administration of NSAIDs in *IL10^−/−^* mice. If observed, this would support and provide a pre-clinical model and mechanistic explanation for the reported worsening of symptoms by NSAIDs in patients with IBD. AIEC is a gut pathobiont found heterogeneously in patients with IBD^11^ and is associated with the pathogenesis of IBD. Consistent with our hypothesis, we show that AIEC colonization determined the severity of NSAID-triggered inflammation in *IL10^−/−^* mice, which was accompanied by activation of the NLRP3 inflammasome, Caspase-8, apoptosis, and pyroptosis, promoting inflammation and cell death.

For this study, we modified a previously reported model of piroxicam-accelerated colitis (PAC)^41^ by pre-colonizing *IL10^−/−^* mice with AIEC followed by 5 days of piroxicam exposure. Using this design, we showed that piroxicam can only induce colitis in mice pre-colonized with AIEC. Colitis did not occur in *IL10^−/−^* mice colonized with AIEC alone or treated with the NSAID alone. The inflammation in the AIEC/piroxicam combination group was accompanied by elevated levels of pro-inflammatory cytokines (e.g., IL-6, TNFα, IFNγ) and infiltration of activated immune cells including T-cells and macrophages. Changes in the colonic epithelial structure (altered tight junction proteins, elongated crypts, crypt abscesses, and goblet cell loss) were also observed in these mice. In contrast to the PAC model,^41^ the conditions for our model were optimized [i.e., a lower dose of piroxicam (100 vs 200 ppm) and fewer days of feeding (5 vs 9 days) were used] to reduce their adverse effects on animal health after antibiotic treatment and AIEC colonization (data not shown). In the PAC model, ulcerations were detected in severe cases, while these were seldom noted in the AIEC/piroxicam combination group. Alterations in the fecal microbiota in AIEC/piroxicam group were subtle compared to untreated mice and to other pre-clinical models such as the dextran sodium sulfate (DSS)-model.^37^ Changes in the experimental design and the less severe impact on epithelial damage in our model compared to, e.g., the DSS-model^34^ might have contributed to the subtle microbial alterations.

Previous studies identified that NLRP3 inflammasome-derived IL-1β release plays a critical role in NSAID-induced enteropathy^20^ and treatment with NLRP3 inhibitors such as MCC950 ameliorates colitis.^42,43^ IL-10 produced from macrophages was also reported to be a negative regulator of NLRP3 when induced by different stimuli.^44^ In our study, we found that, in addition to NLRP3 activation, Caspase-8 was also activated in the AIEC/piroxicam combination group. Previous studies have shown that epithelial deletion of Caspase-8 promoted DSS-induced colitis. This effect was associated with increased levels of RIP3 and a reduced number of anti-microbial Paneth cells, alluding to Caspase-8 regulation of necroptosis and inflammation in the pathogenesis of colitis.^24^ However, no changes in necroptotic cell death markers such as RIPK1, RIP3, or phospho-MLKL were observed in our study in the control or Caspase-8 inhibitor group.

We found that small-molecule inhibitors targeting NLRP3 and Caspase-8 rescued the inflammatory phenotype observed in the AIEC/piroxicam group, indicating an indispensable role of Caspase-8 and NLRP3 in AIEC/NSAID-induced inflammation and in IBD-pathogenesis. Interestingly, NLRP3 inhibition targeted the inflammatory response affecting macrophages leading to an increase in the number of CD206^+^ CD163^+^ M2 macrophages in the spleen and a reduced M1 macrophage gene expression profile in the colon, which is in line with a previous study in a model of valve stenosis and calcification.^45^ No increase in M2 cells and a non-significant reduction in macrophages (CD11b^+^CD14^+^) were seen in the Caspase-8 inhibitor group, indicating potential differences in the regulation of macrophages by these proteins. Both inhibitors significantly reduced the presence of activated T-cells and associated cytokines such as IFNγ and IL-17α and reduced apoptotic and pyroptotic cell death, which probably contributed to the overall ameliorated phenotype presented by either of the inhibitor-treated groups.

Although no significant alterations in microbiota composition or individual SCFAs were detected in the inhibitor-treated groups, an increased abundance of *Clostridium* cluster *XIVa* was seen in both NLRP3 and Caspase-8 inhibitor groups. This is an SCFA producing bacteria, which can regulate Treg cells, and whose abundance is generally decreased in IBD.^46,47^ A similar subtle effect on microbiota composition was reported in a model of experimental autoimmune encephalomyelitis (EAE) after NLRP3-inhibitor (MCC950) administration.^48^ Although no increase in butyrate was noted in either of the inhibitor treatment groups, a higher induction of propionate and total SCFA was observed, potentially indicating the contribution of this genus to SCFA generation and reduced inflammation. The non-significant findings could also reflect the small group size comparisons. For this reason, future studies are warranted to further dissect the changes in microbial taxa following the use of the NLRP3 and Caspase-8 inhibitors.

In conclusion, our findings provide new insights into how NSAID and an opportunistic IBD-associated gut pathobiont can synergize to worsen IBD symptoms and inflammation. To the best of our knowledge, this is the first time that Caspase-8 has been reported as a potential regulator of AIEC and NSAID-induced colitis *in vivo*. Our data suggest that genetic factors alone are not enough to trigger pathogen-like activity by a pathobiont, and other factors such as environmental (exposure to NSAIDs) are needed to trigger a pathobiont’s pathogenic potential. Further, we showed that targeting either Caspase-8 or NLRP3 could be a potential therapeutic strategy for IBD patients with NSAID-worsened inflammation. To date, there is no report in the literature that have examined or correlated the abundance of AIEC in patients with IBD and NSAIDs. We propose that future clinical IBD studies focusing on the role of NSAIDs should consider the status of the colonization of pathobionts such as AIEC as one of the factors influencing the inflammatory phenotype.

## Limitation of study

One of the limitations of this study is the lack of information regarding the cell types in which activation of the NLRP3 inflammasome, Caspase-8, and biochemical markers of apoptosis and pyroptosis occurs. To mechanistically validate the findings from our model, we used human and murine epithelial cell lines (HT29, DLD1, CMT96) and primary cells including murine intestinal crypts and bone-marrow derived macrophages. However, none of these cell systems responded to *in vitro* activation with piroxicam, although previous studies have shown piroxicam-induced cell death in HCT116 and CaCo-2 cells.^49^ With these limitations in mind, future studies are needed to further investigate the molecular and cellular mechanisms underpinning cross-regulation of the Caspase-8/NLRP3 axis. The generation of conditional knock-out mice for Caspase-8 and NLRP3 on an *IL10^−/−^* background would be necessary to identify the cell specificity of Caspase-8/NLRP3 crosstalk and activation. Multiple staining for co-localization of ASC, Caspase-8, and NLRP3 on colon sections would also provide further information on cell-specific activation of these proteins and associated signaling complexes.

## Materials and methods

### Reagents and resources

Providers and catalog numbers of reagents and resources are listed in Table 1 unless otherwise stated.

**Table 1.** Reagent and resources.

Reagent or Resource	Source	Identifier
Antibodies		
Alexa Fluor® 700 anti-mouse CD45 Antibody	Biolegend	Cat# 103128
Brilliant Violet 421™ anti-mouse CD4 Antibody	Biolegend	Cat# 100437
Anti-mouse CD8 PerCP Cy5.5	Biolegend	
APC anti-mouse CD69 Antibody	Biolegend	Cat# 104514
Anti-mouse/human CD11b PECy-7 (clone M1/70)	Biolegend	Cat# 101216
Anti-mouse/human CD11b PE	Biolegend	Cat# 101207
Anti-mouse CD14 BV786	BD Biosciences	Cat# 740883
Anti-mouse I-a/I-E FITC (clone M5/114.15.2)	Biolegend	Cat# 107606
Anti-mouse F4/80 PE-Cy7	Biolegend	Cat# 123113
Anti-mouse CD206 BV785 (clone C068C2)	Biolegend	Cat# 141729
Anti-mouse CD163 BV421 (clone S15049I)	Biolegend	Cat# 155309
Anti-mouse CD16/CD32 (Fc Block clone 2.4G2)	BD Biosciences	Cat# 553142
Rabbit monoclonal NLRP3	Cell signaling technology	Cat# 15101S
Rabbit monoclonal cleaved caspase 8	Cell signaling technology	Cat# 8592S
Rabbit monoclonal cleaved caspase 1	Cell signaling technology	Cat# 89332S
Rabbit monoclonal IL1β	Cell signaling technology	Cat# 31202S
Rabbit monoclonal cleaved IL1β	Cell signaling technology	Cat# 63124S
Rabbit monoclonal IL18	Cell signaling technology	Cat# 57058S
Rabbit monoclonal Cleaved IL18	Cell signaling technology	Cat# 57058S
Rabbit monoclonal caspase 3	Cell signaling technology	Cat# 9662
Rabbit monoclonal cleaved caspase 3	Cell signaling technology	Cat# 9664 T
Rabbit monoclonal cleaved PARP	Cell signaling technology	Cat# 94885
Rat monoclonal caspase 11	Cell signaling technology	Cat# 14340S
Rabbit monoclonal cleaved gasdermin D (GSDMD)	Cell signaling technology	Cat# 10137S
Anti-N4BP1 antibody	Abcam	Cat# ab133610
HRP-linked anti-rabbit IgG	Cell signaling technology	Cat# 7074S
HRP-linked anti-rat IgG	Cell signaling technology	Cat# 7077
HRP-linked anti-goat IgG	Sigma-Aldrich	Cat# SAB3700284
Rabbit anti-Mouse GAPDH IgG	R&D Systems	Cat# AF5718
Rabbit anti-Mouse Tubulin IgG	Cell signaling technology	Cat# 2148
Rabbit anti-Mouse β-actin IgG	Novus	Cat#NB600-532
Bacterial and virus strains		
Adherent-Invasive Escherichia coli Strain HM605	(Martin H. M. et al. 2004)	PMID: 15236175
Chemicals, peptides, and recombinant proteins		
Z-IETD-FMK	MedChemExperss	Cat#HY-101297
MCC950 sodium	MedChemExperss	Cat#HY-12815A
LB Broth	Sigma-Aldrich	Cat#L3022
Ampicillin sodium salt	Sigma-Aldrich	Cat#A9518
Streptomycin sulfate salt	Sigma-Aldrich	Cat#S9137
Sodium bicarbonate	Sigma-Aldrich	Cat#S5761
cOmplete™ Protease Inhibitor Cocktail	Sigma-Aldrich	Cat# 11697498001
Halt Protease Inhibitor Cocktail, EDTA-Free (100X)	ThermoFisher Scientific	Cat#87785
Sodium pyrophosphate dibasic	Sigma-Aldrich	Cat#71501
SensiFAST™ Probe No-ROX	Bioline	Cat#BIO-86050
TURBO DNA-free™ Kit	Invitrogen	Cat#AM1907
Protector Rnase inhibitor	Roche	Cat#3335402001
Transcriptor Reverse Transcriptase	Roche	Cat#3531287001
Sodium Hydroxide (For pH)	Sigma-Aldrich	Cat#567530-250 GM
β-Glycerophosphate disodium salt hydrate	Sigma-Aldrich	Cat#G9422-10 G
DNase I	Sigma-Aldrich	Cat#10104159001
RNase	Sigma-Aldrich	Cat#10109134001
HEPES solution	Sigma-Aldrich	Cat#H0887-20ML
Sodium orthovanadate	Sigma-Aldrich	Cat#450243-10 G
EDTA	Sigma-Aldrich	Cat#03690-100ML
EGTA	Sigma-Aldrich	Cat#03777
Sodium pyrophosphate decahydrate	Sigma-Aldrich	Cat#221368-100 G
Sodium fluoride	Sigma-Aldrich	Cat#201154-100 G
Sodium chloride	Sigma-Aldrich	Cat#S3014-500 G
Triton X-100	Sigma-Aldrich	Cat#93443
Pierce™ BCA Protein Assay Kit	ThermoFisher Scientific	Cat#23225
WesternBright Quantum HRP substrate	Advansta	Cat#K-12042-D10
RNAlater	Sigma-Aldrich	Cat#R0901
Critical commercial assays		
QIAamp Fast DNA Stool Mini Kit	Qiagen	Cat# 51604
RNeasy Mini Kit	Qiagen	Cat# 74106
U-PLEX Biomarker Group 1 (ms) Assays	Mesoscale Discovery	Cat# K15069L-2
Deposited data		
16S rRNA Gene Sequencing	This paper	NCBI: PRJNA849757
Experimental models: Organisms/strains		
Mouse: IL10^−/−^ mice (B6.129P2-Il10tm1Cgn/J)	Biological service unit, University College Cork, Ireland	Charles River Laboratories, UK
Oligonucleotides		
See Table 2 for the full list	N/A	N/A
Software and algorithms		
GraphPad Prism 8	GraphPad	www.graphpad.com
FCS Express V5 software	De Novo Software	https://denovosoftware.com
R Studio (v 3.6.1).	R	https://www.rstudio.com
USEARCH (64 bit; v 8.1)	N/A	https://drive5.com/usearch
ImageJ 1.8	(Schneider et al., 2012)	https://imagej.nih.gov/ij
Other

**Table 2.** Primers and probes used in the study.

Gene	Forward Sequence (5’>3’)	Reverse Sequence (5’>3’)	Probe
Caspase 1	cccactgctgatagggtgac	gcataggtacataagaatgaactgga	103
Caspase 11	tggtcttgtgacttggaggac	agaaacgttttgtcagggtca	105
Il1β	agttgacggaccccaaaag	agctggatgctctcatcagg	38
Il18	caaaccttccaaatcacttcct	tccttgaagttgacgcaaga	46
Nlrp3	cccttggagacacaggactc	gaggctgcagttgtctaattcc	82
Asc	gacatgttgacttctcctgacg	cgacatccagtcagtcatgg	71
Gasdermin D	tgtcaacctgtcaatcaagga	agccaaaacactccggttc	1
Caspase-3	gaggctgacttcctgtatgctt	aaccacgacccgtccttt	68
Klf4	cgggaagggagaagacact	gagttcctcacgccaacg	62
E-cadherin	gctctcatcatcgccacag	gatgggagcgttgtcattg	18
Cxcl2	aaaatcatccaaaagatactgaacaa	ctttggttcttccgttgagg	26
β-Actin	aaggccaaccgtgaaaagat	gtggtacgaccagaggcatac	56
Il17a	gattttcagcaaggaatgtgg	cattgtggagggcagacaat	34
Ccl2	catccacgtgttggctca	gatcatcttgctggtgaatgagt	62
Zo1	tttgagagcaagccttctgc	agcatcagtttcgggttttc	4
Muc2	acctccaggttcaacaccag	gttggccctgttgtggtct	10
Ifnγ	atctggaggaactggcaaaa	ttcaagacttcaaagagtctgagg	21
Caspase 8	tcgttctgatctaagctctcacc	accctcacctggtttctgatt	18
Caspase 3	gaggctgacttcctgtatgctt	aaccacgacccgtccttt	68
Nlrp6	ccagcttctgcatctgagagt	ctcccttgccactgcatc	15
Il6	gctaccaaactggatataatcagga	ccaggtagctatggtactccagaa	6
Inos	gggctgtcacggagatca	ccatgatggtcacattctgc	76
Cxcl10	gctgccgtcattttctgc	tctcactggcccgtcatc	3
Ccl5	gagtggtgtccgagccata	tgcagaggactctgcgacagc	110
Stat 1	tctctagcggatcttctgga	gcagcacaacatacggaaaa	80
Tnfα	tcttctcattcctgcttgtgg	caccccgaagttcagtagaca	49
16S_V3-V4	Tcgtcggcagcgtcagatgtgtataagagacagcctacgggnggcwgcag	gtctcgtgggctcggagatgtgtataagagacaggactachvgggtatctaatcc	

### Bacterial strain and growth

Adherent-invasive *E. coli* HM605 stain was previously isolated from colonic biopsy of CD patients^32^ and was provided by Dr David Clarke, School of Microbiology, UCC. For mouse oral gavage, *E. coli* HM605 was grown by culturing a single colony in 5 mL Luria Bertani (LB) Broth (Cat#L3022, Sigma-Aldrich) with 50 µg/mL ampicillin (Cat#A9518, Sigma) at 37°C overnight without shaking. The next day, the overnight culture was washed twice with PBS (Cat#P4417, Sigma) by centrifugation at 500 g for 10 min. After the second wash, the bacterial pellet was re-dispersed in PBS supplemented with 10% 1 M NaHCO_3_ (Cat#S5761, Sigma) with the final OD_600_ of 5. Each mouse was gavaged with 200 μL of bacterial culture with an OD_600_ of 5.

### Mice

*IL10^−/−^* mice (B6.129P2-Il10tm1Cgn/J) were acquired from Charles River Laboratories, UK, and bred at the Bioscience Service Unit (BSU)-Annex at University College Cork. All animals were housed in individually ventilated cages (IVCs, OptiMICE, UK), with a controlled environment (20–22°C, 12 hours (hr) light:dark cycle) and given food and water *ad libitum*. All animal studies were designed with consideration for the three Rs (Replacement, Reduction, and Refinement) and were approved by the Animal Experimentation Ethics Committee (AEEC; applications #2018-028 and #2020/007) of University College Cork and by the Health Products Regulatory Authority (HPRA, project nrs AE19130/P101 and AE19130/P138).

To establish a model of AIEC-colonization and piroxicam-induced colitis, male *IL10^−/−^* mice (8–16 weeks) were randomized into four groups, PBS (vehicle), AIEC only, piroxicam only, and AIEC and piroxicam combination. To establish colonization of AIEC, *IL10^−/−^* mice were given streptomycin (5 g/L, Cat#S9137, Sigma-Aldrich) in the drinking water *ad libitum* for 24 hr.^50,51^ After streptomycin treatment, mice were orally gavaged with 10^8^ to 10^9^ colony forming units (CFU) of Adherent-invasive *E. coli* HM605 in 0.2 mL/mouse. Three days after AIEC infection, mice were given 100 parts per million (ppm) of piroxicam (Sigma) homogenized in regular chow (Envigo, UK) for up to 5 days followed by 9 days of regular chow. At day 14, mice were sacrificed by cervical dislocation and intestinal tissue was collected for further analysis (Figure 1a). Fecal samples were collected at days 0 and 14 for 16S rDNA analysis (Figure 1a).

For the inhibitor experiment, the NLPR3 inhibitor MCC950^38^ (20 mg/kg, Cat#HY-12815A, MedChemExperss) and Caspase-8 inhibitor Z-IETD-FMK^39^ (10 mg/kg, Cat#HY-101297, MedChemExperss) were diluted in DMSO (Cat# D2650, Sigma) and injected, intraperitoneally, in a 0.2 mL volume, every other day starting from day −5 (Figure 2a) followed by streptomycin treatment, AIEC gavage, and piroxicam feeding as outlined. Control mice were injected with DMSO in the same manner as the inhibitors. Mice were sacrificed on day 14 (Figure 2a). Intestinal tissue, spleen, mesenteric lymph nodes (MLNs) and cecal samples and blood were collected at sacrifice for further analysis. Fecal samples were collected on days 0 and 12 of the study for 16S rDNA analysis (Figure 2a).

### Collection of samples

Colons were excised, opened longitudinally, and washed in PBS. Colon length was recorded, and colon was divided into two pieces with 3 cm of distal colon (weighed) and divided into two longitudinal pieces; one was snap frozen in liquid nitrogen for western blot analysis, and the other section was rolled, embedded in optimum cutting temperature (OCT, Cat#4583, Tissue-Tek™) compound and snap frozen in liquid nitrogen for histological analysis. For RNA isolation and RT-qPCR, a 0.5 cm piece of the most distal colon was stored in RNAlater (Cat#3335402001, Roche) for 24 hr at 4°C and snap frozen in liquid nitrogen. The spleen and MLNs were removed and processed for isolation of cells (see below). Feces and cecal content were collected and snap frozen in liquid nitrogen for 16S rDNA analysis. All collected samples were stored at −80°C until needed.

### Hematoxylin and Eosin and PAS staining and scoring

OCT-embedded distal colon sections were cut in Leica CM 1950 Cryostat at 3–5 µm and fixed in 10% Neutral Buffered formalin, followed by hematoxylin and eosin (H&E) staining as previously reported.^52^ For the detection of mucus producing cells, sections were fixed in 100% Iso-propyl alcohol, stained with Alcian blue and periodic acid-Schiff (PAS) as previously reported.^53^ H&E-stained samples were scored according to^41^ with some modifications. Briefly, inflammation (0–4), hyperplasia (0–4), and extent of area involved (0: 0–10%; 1: 10–25%; 2: 25–50%; 3: 50–75%; 4: 75–100%) were scored individually resulting in a total score of 12. No areas of ulcerations were observed in the sections. Pictures were taken with a Leica DMLB microscope at 10x zoom.

### Bacterial counts

To enumerate the bacteria in the stool samples, fecal pellets were weighed and homogenized in PBS at a concentration of 0.1 mg/mL. Homogenized samples were plated onto LB agar with 50 µg/mL ampicillin. After culturing at 37°C overnight, bacterial counts were recorded.

### Real time qPCR analysis

Total RNA from the colonic tissue was isolated using the Qiagen RNeasy kit (cat #74104 Qiagen) following the manufacturer’s protocol and quantified using a NanoDrop Spectrophotometer (ND1000). The RNA was treated with Turbo DNA-free kit (Invitrogen), to remove genomic DNA, following the manufacturer’s instructions. 1 μg of RNA was reverse transcribed using Transcriptor Reverse Transcriptase from Roche. The qPCR was carried out using a Roche LightCycler 480 instrument and SensiFAST No-ROX mix. The Ct values obtained were compared using 2-ΔCt. The expression of genes was normalized to that of β-actin. Primers and probes were designed using Universal Probe Library Assay Design Center (https://www.roche-applied-science.com/sis/rtpcr/upl/adc.jsp; Roche Applied Science). Primer and probe sequences of all genes analyzed are summarized in Table 2.

### Meso scale discovery (MSD) multiplex assay for cytokine protein quantification

Colonic tissue was homogenized as previously described.^52^ Briefly, colon tissue was homogenized in 350 µL of lysis buffer (40 mL of PBS), 10% FCS (cat nr# F9665, Sigma) 2 Complete Protease Cocktail Inhibitor Tablets (Roche, Mannheim, Germany). Each sample was subject to three rounds of homogenization using a MagnaLyser (Roche) at 6,000 rpm for 15 seconds, placing the tubes on ice for 30 seconds between each round, followed by centrifugation at 10,000 g for 10 minutes at 4 °C. The supernatants/homogenates were aliquoted into fresh 1.5 mL tubes and stored at −80 °C until use. 25 µL of lysates per sample were used in MSD U-plex assay to quantify the cytokines IFNγ, IL-1β, IL-6, and mKC according to the manufacturer’s instructions. Briefly, U-plex 10 assay plate was coated with linker-coupled biotinylated capture antibody overnight at 4°C. Next day, the plate was washed thrice with PBS containing 0.1% tween-20 (Cat# 1379 Sigma). After washing, samples and standards were added to the plate and incubated at room temperature (RT) for 1 hr with shaking, followed by three washes with PBS containing 0.1% tween-20 (PBST). Fifty microliters of secondary detection antibodies solution were added for 1 hr at RT with shaking following PBST washes. After the secondary incubation, the plate was washed thrice with PBST, followed by 150 µL of read buffer. The plate was read immediately after adding read buffer using MESO QuickPlex SQ 120. Data graphed as cytokine levels are expressed as pg cytokine/mg colonic tissue.

### Western blot analysis

Western blot was performed as described with some modifications.^54^ Briefly, colonic tissue samples were homogenized in tissue lysis buffer (50 mM NaCl, 50 mM NaF, 50 mM Na_4_P_2_O_7_, 5 mM EGTA, 5 mM EDTA, 2 mM Na_3_VO_4_, 10 mM HEPES, 1% Triton X-100, pH 7.4) supplemented with 1 × Halt Protease and Phosphatase Inhibitor Cocktail (Cat# 78440, Thermo Scientific), 0.02 mg/ml RNase, 0.2 mg/ml DNase, 0.01 mM Na_3_VO_4_, 0.005 mM Na_4_P_2_O_7_, 0.01 mM β-glycerophosphate. Lysate was cleared by centrifugation for 15 min at 14,000 rpm and 4 ℃, and protein concentration was measured using Pierce BCA Protein Assay Kit (Cat# 23225, Thermo Scientific). Forty to eighty micrograms of protein samples were denatured in 1 × Bolt LDS Sample Buffer (Invitrogen) supplemented with 1 × Bolt Sample Reducing Agent (Invitrogen) by heating for 10 min at 75 ℃. Samples were separated on Bolt 4–12% Bis-Tris Plus Gels (Invitrogen) and transferred to a PVDF membrane (Cat# IPVH00010, Millipore) using the Mini Blot Module (Invitrogen). The membrane was blocked with 5% Tris buffer saline (TBS) with 0.1% Tween-20 (TBST) for 1 hr, at RT and incubated with the primary antibody overnight at 4°C. Next day, the membrane was washed with TBST and probed with the secondary antibody for 1 hr, at RT followed by TBST wash and detection with WesternBright Quantum HRP substrate (cat no. K-12042-D10, Advansta) and LAS-3000 Imager (Fujifilm) and processed with ImageJ software (without gel splicing and brightness/contrast adjustment). Antibodies used for western blotting are listed in Table 1.

### Flow cytometric analysis

Spleens were removed, and single-cell suspensions were prepared as reported with some modifications.^55^ Briefly, to isolate cells from the spleen, the tissue was pressed through a 100 μm cell strainer (Sarstedt, Germany) positioned on a 50 mL tube using the plunger of a 1 mL syringe and washing the strainer with 1X sterile PBS supplemented with 1% FCS. Samples were centrifuged for 5 min at 300 × g and red blood cells were lysed by 10 min incubation on 37℃ with 5 mL of 1 X RBC lysis buffer (eBioscience). Immune cells were re-suspended in 1X PBS supplemented with 1% FCS. Isolated cells were washed three times in PBS supplemented with 1% bovine serum albumin (BSA, Sigma) and 0.1% sodium azide (Sigma). Nonspecific binding of antibodies (Abs) to Fc receptors was blocked by pre-incubation of cells with monoclonal Abs (mAb) 2.4G2 directed against the FcgRIII/II CD16/CD32 (Table 1) (0.5 ng mAb per 10^6^ cells). 1 × 10^6^ cells were incubated with 0.5 ng of the relevant mAb for 20 min at 4°C and washed twice. mAbs used in this study are listed in Table 1. Data were analyzed using FCS Express V5 software (De Novo). Cells were analyzed using the three laser (405 nm, 488 nm, 460 nm) BD Celesta FACS Analyzer. The forward narrow angle light scatter was used as an additional parameter to facilitate the exclusion of dead cells and aggregated cell clumps. A forward scatter height (FSC-H) vs forward scatter area (FSC-A) density plot was used to exclude doublets. Then, FSC-A vs side scatter area (SSC-A) density plot was used to identify cells of interest (Figure S3A-C). For T cell analysis, CD4 vs CD8 dot plots were obtained after gating cells with CD45 expression (Figure S3A). For macrophage analysis, CD11b vs CD14 and CD11b vs F4/80 dot plots were obtained by gating on cells with CD45 expression (Figure S3B). CD206 vs CD163 dot plots were obtained after gating on CD11b^+^F4/80^+^ cells (Figure S3C).

### DNA extraction, 16S rRNA gene sequencing, and gut microbiota analyses

DNA from fecal samples were extracted using a QIAamp Fast DNA Stool Mini Kit (Qiagen Cat# 51604) following the manufacturer’s instructions. Amplicon sequencing of the 16S rDNA V3-V4 region of bacterial communities was performed as previously described,^56^ utilizing 2x250bp paired-end Illumina MiSeq chemistry (Genewiz; Leipzig, Germany). Raw sequencing reads were processed using an in-house 16S rRNA processing pipeline, employing USEARCH (64 bit; v 8.1). Briefly, paired-end reads were merged and filtered using a < 0.5 expected error rate per nucleotide and total length. Reads were dereplicated, and singletons removed, following the trimming of the forward and reverse primers (“-stripleft 17” and “-stripright 21”, respectively). Operational taxonomic units (OTUs) were clustered at 97% identity, and reference-based chimera removal was performed using UCHIME. OTUs were assigned taxonomic information by aligning reads to the RDP Gold database using the RDP Classifier (v 2.12).

The 16S rRNA reads assigned taxonomic information was converted into a count matrix and imported into R Studio (v 3.6.1). The 16S data was analyzed using the metadata accompanying the relevant mouse trials and the RT-qPCR. The 16S rRNA OTU reads aligned per OTU were converted into relative abundances using the “funrar” package.^57^ Dataframes and matrices were manipulated as necessary using the “reshape2” package.^58^ Publication quality images were generated using the “ggplot2” and “ggpubr” packages.^59,60^ Boxplots represent the standard Tukey representation, with boxes representing the 25th, 50th (median) and 75th interquartile range (IQR) percentiles and the whiskers encompassing values within 1.5× the IQR. The specific values of each boxplot are overplotted as opaque gray circular points using the ggplot “geom_jitter” function, whereas boxplot outliers are represented as solid square points.

The alpha- and beta-diversity of the samples were calculated using the R packages “vegan” and “phyloseq”.^61,62^ All alpha-diversity values presented use Simpson’s index, while beta-diversity separation was performed using Canberra distances with PCoA ordination. A color palette for the composition bar plots was obtained through the “pals” R package.^63^ The log_2_ fold change of epithelial and inflammatory biomarker genes, and 16S rRNA taxa, was performed using the “DESeq2” package after scaling values to integers.^64^ Correlations between microbial taxa and epithelial and inflammatory biomarker genes were performed using base R’s “stats” package,^65^ with plots generated using the “corrplot” package.^66^ Individual images were manipulated into their final multipanel display using Inkscape (v 1.1.2).

### Short chain fatty acids (SCFAs)

Analysis of SCFAs was performed as recently reported.^67^ Briefly, cecal samples were weighed and diluted 1:10 (w/v) in sterile HPLC grade water. The SCFA-containing supernatant was filtered through 0.2 µm pore size cellulose acetate membrane (GyroDisc CA; Orange Scientific, Braine-l’Alleud, Belgium) and stored at −20°C until HPLC analysis. Quantification of SCFAs in cecal samples was carried out using an external calibration standard curve method.^67^ Cecal SCFA concentrations were expressed as mean µmol per gram wet weight cecum using the following equation: Cecal SCFA (µmol/g) = [organic acid in cecal contents (mmol/mL) × Vd (ml) × 1000]/wet weight cecum (g), where Vd = total volume of dilution.

### Statistical analysis

GraphPad Prism software was used to perform statistical analyses for all data sets except 16S amplicon data. All data were expressed as mean ± SEM One-way ANOVA with Bonferroni test or Kruskal–Wallis with Dunn’s multiple comparison test was used to test for significant differences between various groups. A p-value <0.05 was considered significant.

For analyzing the 16S amplicon data of the relevant mouse trials, the following statistical tests were performed. Wilcoxon tests were performed for nonparametric two-group comparisons, with the p-values presented above the relevant alpha-diversity box plots. Statistical significance in the beta-diversity of mice with respect to days post piroxicam, treatment group, or their interaction was assessed using the “adonis” function of the vegan R package, which performs a permutational multivariate analysis of variance (PERMANOVA) test. The variance (R^2^) and p-values of PERMANOVA tests accompany the relevant PCoA plots. Statistically significant changes in microbial taxa with reference to mice treatment groups were calculated using Wilcoxon tests with Benjamini–Hochberg false discovery rate (fdr) correction. All correlations between microbial taxa and epithelial and inflammatory biomarkers were conducted using Spearman rank correlations.

## Supplementary Material

Supplemental Material

## Data Availability

Raw sequence data from mouse fecal samples have been deposited in the NCBI SRA database under the Bioproject ID PRJNA849757 (https://www.ncbi.nlm.nih.gov/bioproject/PRJNA849757). This paper does not report any original code. Any additional information required to reanalyze the data reported in this paper is available from the lead contact upon request.
