# Supplementary material for: An IBD-associated pathobiont synergises with NSAID to promote colitis which is blocked by NLRP3 inflammasome and Caspase-8 inhibitors
Source: Gut Microbes. 2023 Jan 19;15(1):2163838. doi: 10.1080/19490976.2022.2163838 (PMC9858430; doi:10.1080/19490976.2022.2163838)
Supplement: Supplemental Material [file KGMI_A_2163838_SM7286.zip › Singh et al Supplemental information.docx]

# SUPPLEMENTAL INFORMATION

##
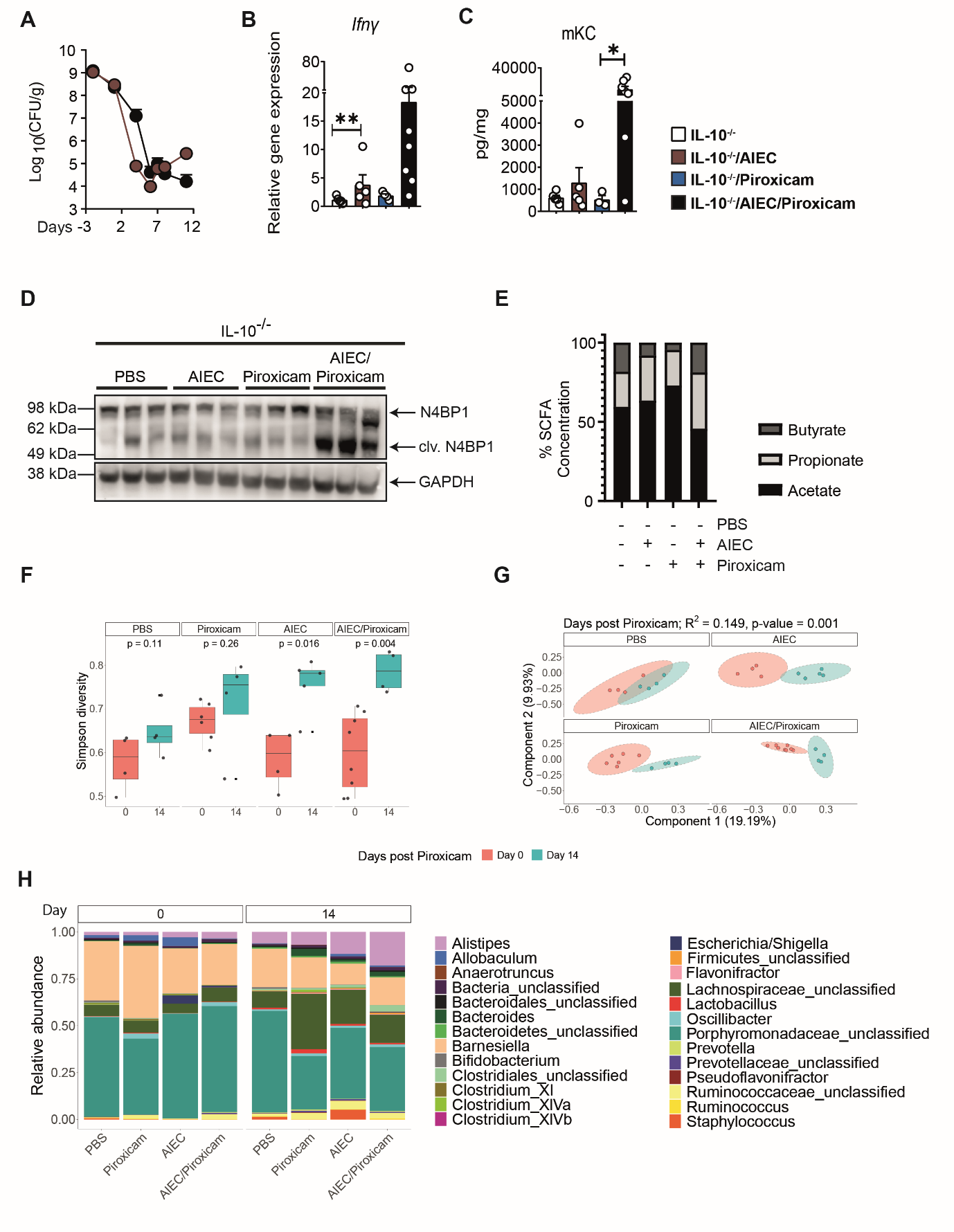


## Figure S1. AIEC colonisation and piroxicam treatment subtly altered the gut microbiota composition

(A) Faecal AIEC colonisation. n=5-8/group. (B) RT-qPCR of colonic *Ifng*. (C) Colon mKC protein. (D) Western blot for N4BP1. (E) Caecal SCFA levels. (F-G) Faecal samples were collected for 16S rRNA analysis at days 0 and 14. (F) Simpson diversity graphed by treatment. (G) PCoA analysis. (H) Faecal bacteria relative-abundance at the genus-level. Microbiota analysis, n=4-9/group. In (A-D), data are presented as mean ± SEM. Significance was determined using one-way ANOVA with Bonferroni or Kruskal-Wallis test with Dunn’s multiple comparison test, respectively. * p < 0.05; ** p <0.01, *** p <0.001.

##
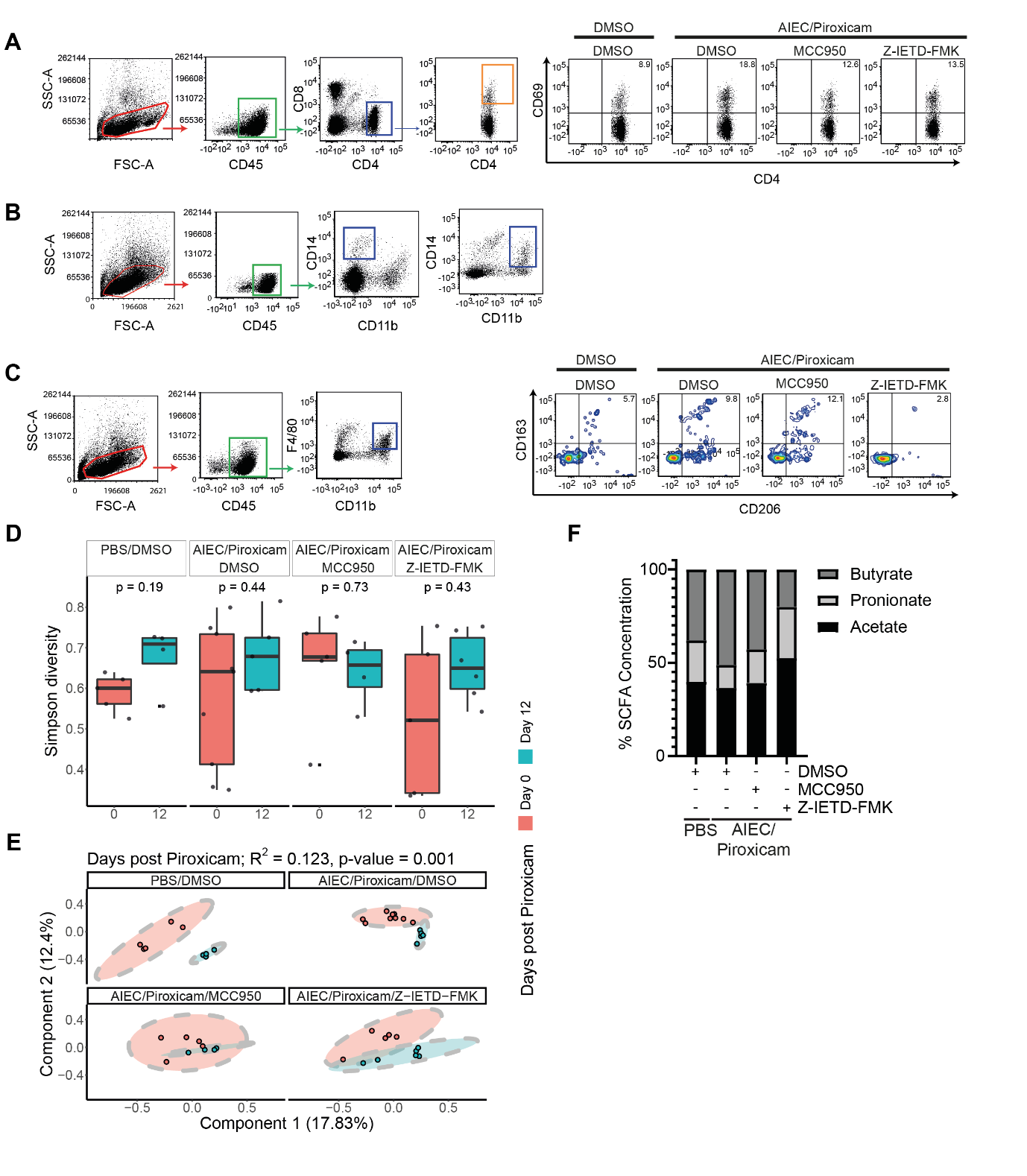


## Figure S2. Impact of Caspase8 and NLRP3 inhibition on T cells and macrophage populations and microbiota composition

(A-C) Gating strategy. Spleen activated T-cells (CD4+ CD69+) and macrophages (CD45+ CD11b+ CD14+; CD45+ CD11b- CD14+; CD45+ CD163+ CD206+ M2 macrophages) were immunophenotyped by flow cytometry. (D) Simpson diversity graphed by treatment. (E) PcoA analysis. (F) Caecal SCFA levels. n=4-7/group. Data are presented as mean ± SEM. Significance was determined using one-way ANOVA with Bonferroni or Kruskal-Wallis test with Dunn’s multiple comparison test, respectively. * p < 0.05; ** p <0.01; ***p <0.001.
