# Supplementary figures and images for: An IBD-associated pathobiont synergises with NSAID to promote colitis which is blocked by NLRP3 inflammasome and Caspase-8 inhibitors
Source: Gut Microbes. 2023 Jan 19;15(1):2163838. doi: 10.1080/19490976.2022.2163838 (PMC9858430; doi:10.1080/19490976.2022.2163838)

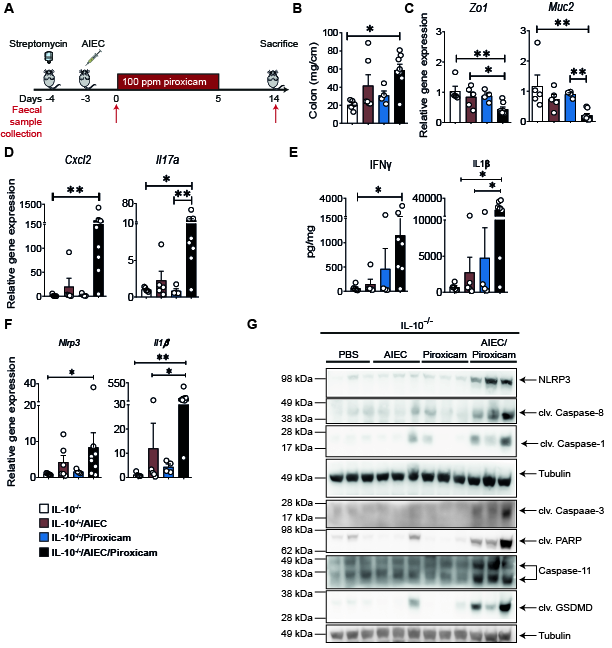

Supplement: Supplemental Material [file KGMI_A_2163838_SM7286.zip › Singh et al Fig1 revised.tif]

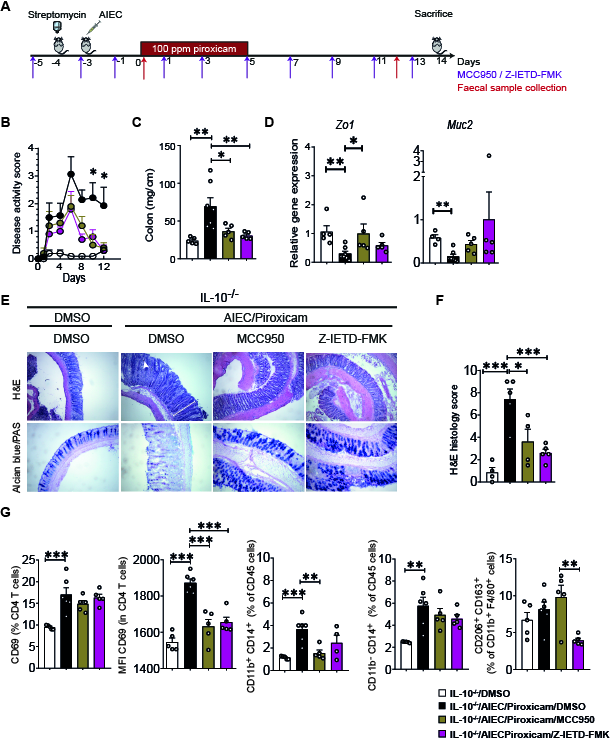

Supplement: Supplemental Material [file KGMI_A_2163838_SM7286.zip › Singh et al Fig2 revised.tif]
